# Beyond biomarkers: Exploring the diverse potential of a novel phosphoprotein in lung cancer management

**DOI:** 10.1111/jcmm.70077

**Published:** 2024-09-20

**Authors:** Amirabbas Rostami, Ehsan Ranjbar, Somayeh Amiri, Fatemeh Ezzatifar

**Affiliations:** ^1^ Department of Internal Medicine, School of Medicine Isfahan University of Medical Sciences Isfahan Iran; ^2^ Department of Urology, Uro‐Oncology Research Center Tehran University of Medical Sciences Tehran Iran; ^3^ Department of Microbiology, Faculty of Advanced Science and Technology, Tehran Medical Sciences Islamic Azad University Tehran Iran; ^4^ Department of Immunology, School of Medicine Mazandaran University of Medical Sciences Sari Iran

**Keywords:** autoantibody, diagnosis, lung cancer, nucleolin, prognosis, therapy

## Abstract

In addressing the challenges of lung cancer, attention has increasingly turned to molecular diagnostics and targeted therapies, with nucleolin (NCL) assuming a pivotal role, especially in non‐small cell lung cancer. The aberrant activity and cellular distribution of NCL act as crucial biomarkers for early detection and treatment monitoring, showing a strong correlation with disease progression and patient prognosis. Elevated NCL levels signal advanced disease and poorer outcomes, underscoring its significance in evaluating disease severity and therapeutic response. Strategies targeting the molecular interactions of NCL have spurred innovative approaches to inhibit tumour growth, overcome drug resistance and improve treatment efficacy. These advancements are paving the way for personalized therapies tailored to the unique molecular profiles of patients' tumours. Consequently, NCL stands at the forefront of lung cancer management, symbolizing the move towards more precise and individualized oncology care, and marking substantial progress in therapeutic development.

## 1 BACKGROUND

Orrick et al. (1973) first characterized nucleolin (NCL)^1^ as a critical player in eukaryotic cells, influencing cellular proliferation, DNA replication, apoptosis, ageing, chromatin structure, ribosome production and RNA synthesis.^2^ Intriguingly, in vitro studies have revealed that NCL undergoes self‐cleavage in dormant cells, yet remains intact in actively dividing malignant cells.^3^ Notably, increased levels of NCL mRNA and membranous protein have been observed in various cancers, despite no detected mutations or alternative splicing of NCL gene.^2^ In lung cancer, changes in NCL expression have garnered significant scientific attention due to its association with proteins involved in cancer pathogenesis.^4^ Lung cancer mainly manifests as non‐small cell lung cancer (NSCLC), including adenocarcinoma, squamous cell carcinoma and large cell carcinoma, making up 85% of cases. Small cell lung cancer (SCLC), known for rapid progression and a strong link to smoking, represents a smaller but significant portion. Other forms like mesothelioma, linked to asbestos, and rare cancers like carcinoid tumours also contribute to the spectrum.^5^ Generally, the prognosis of lung cancer depends on its stage and subtype, with treatments such as surgery, chemotherapy and radiation, which often have non‐specific and potentially fatal side effects.^6^

**FIGURE 1:**
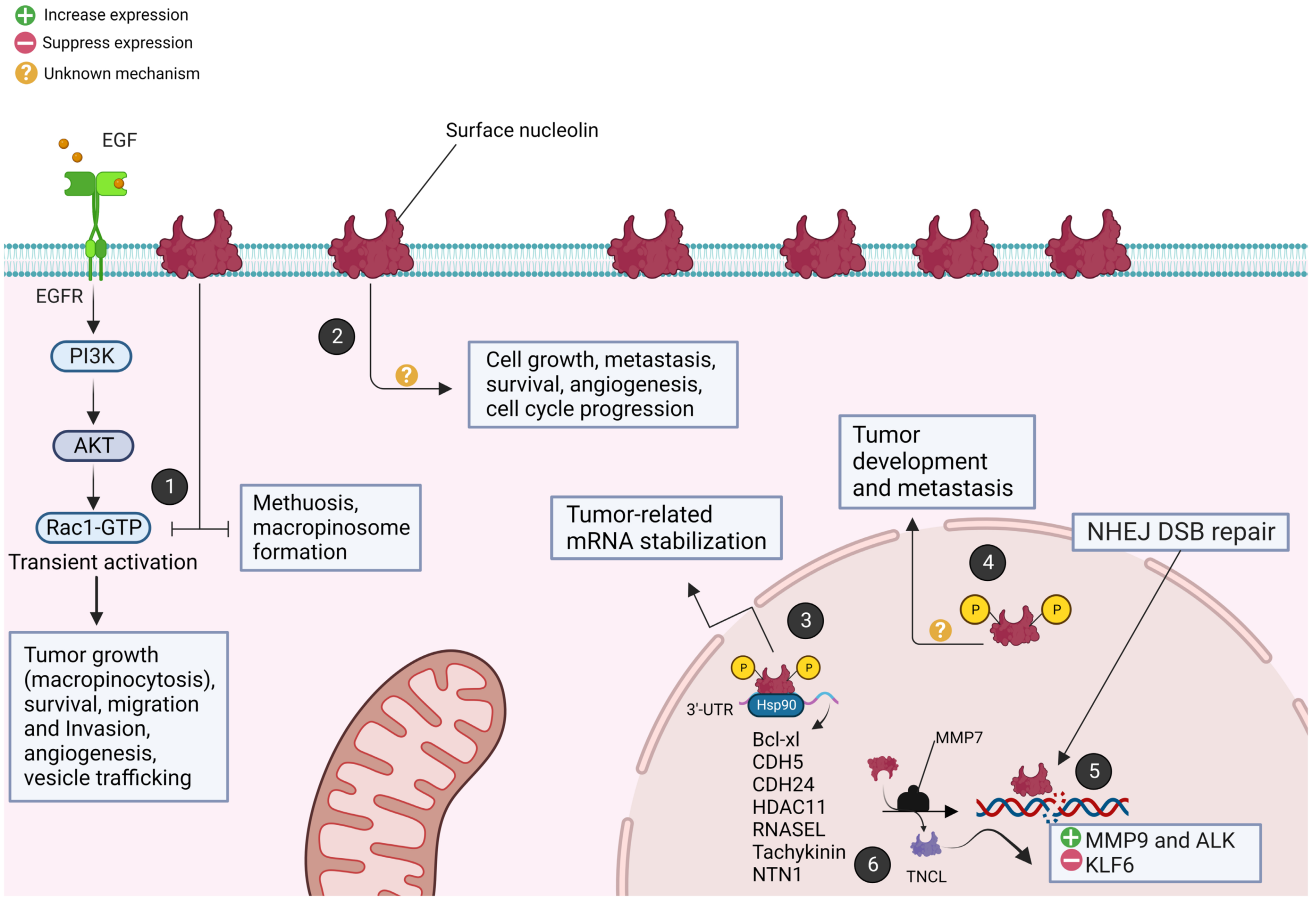
Speculated roles for nucleolin (NCL) in the development of lung cancer. (1) The NCL serves as a negative regulator of Rac1, causing its transient activation in response to growth factor signals like epidermal growth factor (EGF) and others. Thus, the subsequent signalling cascades promote cell growth and survival, cell migration and invasion, angiogenesis and increased vesicle trafficking. Furthermore, NCL overexpression acts as a tumour/metastasis mediator by blocking macropinosome formation and avoiding Rac1‐induced methuosis. (2) On the contrary, through unknown downstream mechanisms, surface NCL stimulates cell proliferation, survival, metastasis, angiogenesis and cell cycle progression. (3) Also, the amplification of tumour‐related mRNAs, including as Bcl‐xl, cadherin‐5 (CDH5), CDH24, histone deacetylase (HDAC)11, tachykinin receptor 1 (TACR1) and netrin 1 (NTN1), occurs during mitosis due to the stabilization of nuclear NCL‐tumour mRNA complexes by Hsp90. (4) Though downstream pathways are unclear, the increased phosphorylated NCL also promote tumour growth and metastasis. (5) To facilitate the replication of the tumour genome and the continuation of the cell cycle, NCL may also promote the non‐homologous end joining (NHEJ) double‐strand DNA breaks (DSB) repair pathway. (6) Also, the matrix metalloproteinase (MMP)7 enzyme cleaves NCL, producing truncated NCL (TNCL), which promotes the production of MMP9 and anaplastic lymphoma kinase (ALK) oncogenes while suppressing the expression of the kruppel‐like factor 6 (KLF6) tumour suppressive gene.

In lung cancer, the stabilization of NCL contributes to carcinogenesis by enhancing the expression of various oncogenic mRNAs.^7^ It is also crucial in forming complexes with c‐Jun that bind to the cPLA2alpha promoter, essential for NSCLC cell proliferation.^8^ Additionally, NCL maintains radiosensitivity in NSCLC by modulating DNA‐dependent protein kinase catalytic subunit (DNA‐PKcs) activity, key in DNA repair^9^ (Figure 1). Recent reports have identified NCL as a crucial hub gene through protein–protein interaction (PPI) network analysis, revealing its significant overexpression in lung adenocarcinoma (LUAD) tissues. NCL alters the tumour microenvironment via the midkine (MDK)‐NCL pathway, leading to the activation of cancer‐associated fibroblasts and subsequently enhancing tumour invasion.^10^, ^11^ Moreover, studies have correlated higher tumour sizes in human lung cancer tissues with increased NCL expression.^12^ Research indicated that NCL expression, alone or with other cell surface markers, associated with patient survival rates and metastasis in certain lung cancers.^12^, ^13^ Also, NCL‐targeting ligands showed significant promise in detecting and treating lung tumours through various in vitro and in vivo studies.^14^, ^15^ These advancements highlight the ongoing search for more effective, targeted therapies, setting the context for exploring the role of NCL within this diverse therapeutic arsenal. So, this review aims to consolidate the current understanding of NCL surface expression, highlight its pivotal role in lung cancer tumorigenesis and diagnosis, as well as assess its potential as a prognostic and therapeutic marker.

## 2 MALIGNANT STRATEGIC RECONFIGURATION OF CELLULAR NCL

Although findings are limited to lung cancer, multiple factors seem to drive the strategic repositioning of NCL from the nucleus to the cytoplasm and plasma membrane, subsequently exploiting its functional capabilities to favour malignant transformations.

### 2.1 Nuclear localization

NCL is a ubiquitously expressed protein predominantly located in the nucleolus, specifically within the dense fibrillary component (DFC), where it is essential for ribosomal DNA (rDNA) transcription. Its accumulation in the nucleus and nucleolus is driven by its bipartite nuclear localization signal (NLS), RNA‐binding domain (RBD) and Arg‐Gly‐Gly (RGG) domains, which bind to rRNA and other nucleolar components. NCL promotes rRNA synthesis and ribosome assembly, vital for rapid cell proliferation and tumorigenesis.^2^ Although over 90% of NCL is in the nucleolus, it also plays significant roles in the nucleus, constituting less than 5% of the total cellular NCL.^16^ In the nucleus, NCL is crucial for transcriptional regulation, mRNA stability and DNA metabolism. Under stress conditions like heat shock and radiation, NCL redistributes from the nucleolus to the nucleus in a p53‐dependent manner, impacting DNA replication and repair.^17^ Also, nuclear NCL regulates oncogene expression by interacting with specific miRNAs, promoting tumour growth.^18^ Additionally, it is vital for transcriptional activities of RNA polymerases I and II, facilitating cell proliferation.^2^, ^19^ It binds to the promoter of vascular endothelial growth factor (VEGF), thereby increasing its expression and promoting angiogenesis.^20^ Nuclear NCL also, interacts with transcription factors like TBX3, aiding in tumour growth and cell migration.^21^ Furthermore, these NCLs are integral to DNA repair and replication processes and are implicated in mechanisms of drug resistance.^22^

### 2.2 Cytoplasmic trafficking

As a dynamic protein, NCL shuttles between the nucleus and cytoplasm, facilitating the transport of ribosomal proteins and its subunits.^23^ Its localization is regulated by phosphorylation: dephosphorylated cdc2 sites promote nuclear localization, whereas phosphorylated sites enhance cytoplasmic accumulation.^24^ Additionally, the C‐terminal domain's post‐translational modifications are vital for its shuttling activity. Laminin, an extracellular matrix protein, significantly influences NCL's localization: in laminin‐coated environments, NCL is primarily nuclear, whereas it shifts to the cytoplasm in the absence of laminin.^25^ Cytoplasmic NCL is also involved in internalization processes, appearing in vesicles associated with EEA1 and lactoferrin. NCL's association with the actin cytoskeleton underscores its importance in endocytosis, as it redistributes between the membrane and cytoplasm.^26^ In the cytoplasm, NCL also regulates the translation of p53 mRNA and protecting tumour cells from apoptosis by stabilizing Bcl‐2 mRNA.^27^ The cytoplasmic NCL is critical for the centrosome cycle, with its absence leading to cell growth arrest and centrosome duplication errors, contributing to cancer progression.^28^ Furthermore, arachidonic acid‐induced phosphorylation causes NCL to accumulate in the cytoplasm, where it co‐localizes with RhoA, indicating a role in tumour metastasis.^29^

### 2.3 Membrane redistribution

Despite lacking a hydrophobic sequence or a specific plasma membrane targeting sequence, NCL is surprisingly found on the cell membrane.^30^ Surface NCL comprises approximately 5%–10% of the total NC present in various cell types.^31^ Membranous NCL interacts with multiple molecules that play crucial roles in cell differentiation (laminin‐1), leukocyte adhesion and trafficking (CD62P and CD62L), inflammation (Factor J), vascularization and tumorigenesis.^32^ It is hypothesized that the translocation of NCL to the plasma membrane occur through a secretory pathway facilitated by various factors.^33^ Growth factors like VEGF can induce the localization of NCL to the cell surface through PI3K‐dependent pathways.^33^ Additionally, N‐glycosylation may influence the expression and functionality of surface NCL.^34^ On the cell surface, NCL is implicated in several key signalling pathways that regulate cell proliferation, cell cycle progression and apoptosis. Moreover, NCL has been found to co‐localize with Fas on the membranes of tumour cells, inhibiting apoptosis by blocking Fas–FasL binding.^35^ Furthermore, NCL promotes cell proliferation by interacting with Ras, activating the Ras/MAPK signalling pathway.^36^ Proteins involved in tumorigenesis and angiogenesis, such as hepatocyte growth factor (HGF), VEGF, MDK, pleiotrophin, urokinase, lactoferrin and tumour necrosis factor‐alpha inducing protein (Tipα), interact with surface NCL, highlighting its potential as a biomarker for cancer diagnosis and a target for therapeutic intervention.^37^

## 3 NCL AND LUNG CANCER DIAGNOSIS

**FIGURE 2:**
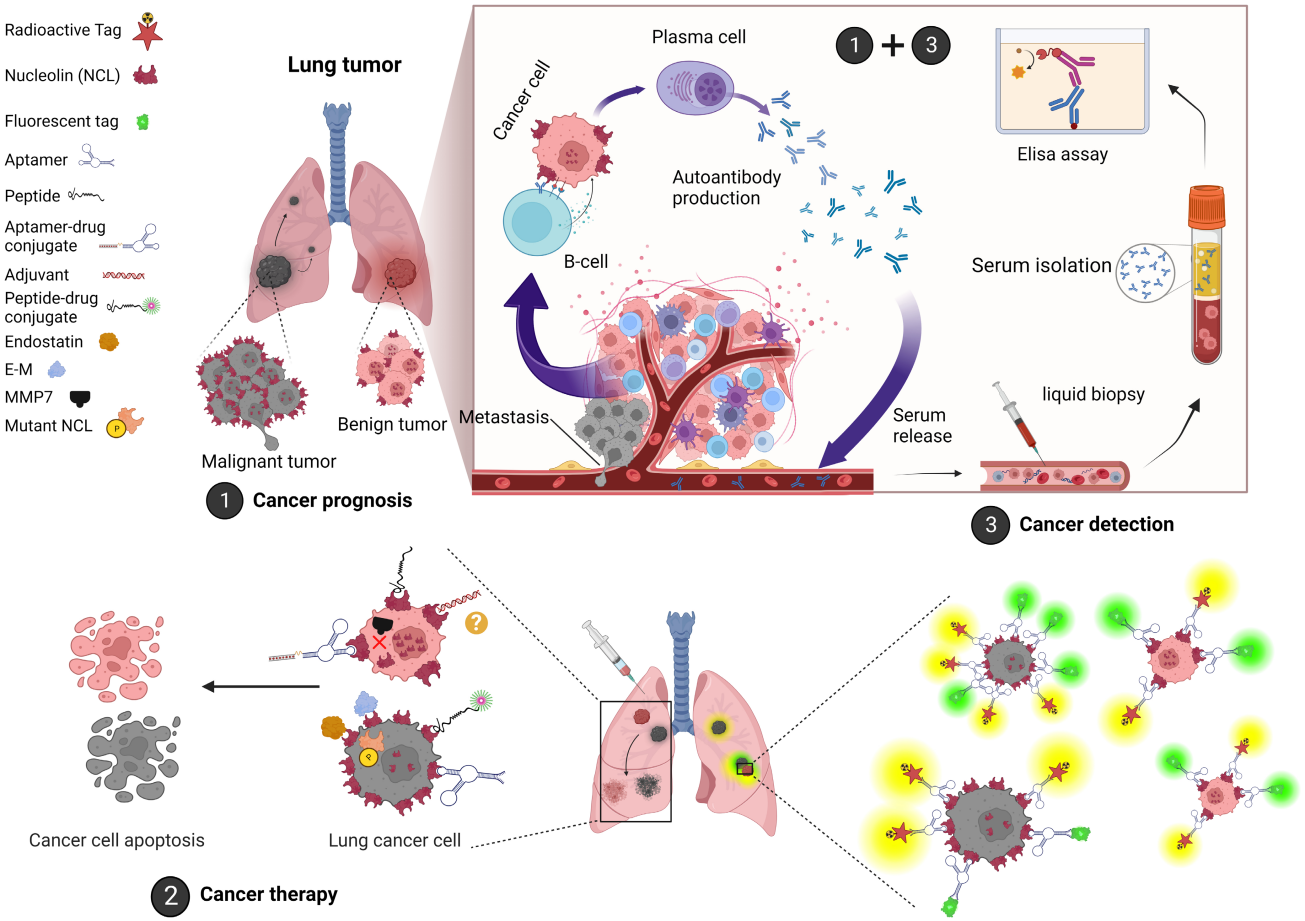
The tripartite role of nucleolin (NCL) in lung Cancer. (1) Elevated NCL expression on the surface of lung tumour cells is associated with grim prognosis. (2) Anticancer ligands like aptamers and peptides, or drug‐ligand conjugates targeting NCL, are being explored for lung tumour therapy. High‐affinity molecules like endostatin show promise in reducing tumour growth. Nucleic acid‐based adjuvants may improve immune detection of lung tumour cells by targeting surface NCL. (3) The third avenue involves attaching therapeutic ligands, like aptamers, to tracing agents such as fluorescent markers and radioactive isotopes, aiming to improve the identification of lung cancer cells. (1) + (3) Conversely, externalized NCL on the cell surface may activate B lymphocytes, generating autoantibodies. Isolating these autoantibodies offers a potential method for early lung cancer screening and insights into tumour proliferation, disease grade and progression.

Despite various developments in diagnostic methods, the pronounced expression of NCL in lung tumour cells and tissues remains a key focus for numerous investigators. Utilizing nanoimaging probes and oligonucleotide (ODN) ligands with strong affinity for NCL, they have successfully identified lung cancer cells directly or indirectly. These ODNs, particularly aptamers, are RNA or DNA strands known for their high specificity, affinity to targets and resistance to degradation by body nucleases.^38^, ^39^ One well‐known NCL aptamer, AS1411, is guanine‐rich and has been tagged with various radioisotopes like ^64^Cu, yielding promising positron emission tomography results both in vitro and in vivo.^14^, ^40^, ^41^ Similarly, a study has demonstrated the fabrication of Cu_2_O nanocubes engineered with the aptamer AS1411. These nanocubes function as surface‐modulated catalytic optical sensors, exhibiting outstanding sensitivity and specificity in the detection of lung cancer cells. Notably, they were capable of identifying as few as 20 cells in actual human serum samples.^42^ In a more advanced application, AS1411 has been integrated with another aptamer and an Arg‐Gly‐Asp (RGD) probe to form SMART (Simultaneous Multiple Aptamers and RGD Targeting) cancer master probes. These probes have demonstrated increased selectivity and diagnostic sensitivity for A549 lung cancer cells in recent study.^43^ Furthermore, researchers have employed molecular identification probes based on DNA sequences capable of forming G4 structures. One such innovative probe, fluorescently tagged and designed to bind to the G4 ligand PhenDC3, mirrors the structure found in tumorigenic miR‐150 and effectively identifies NCL‐positive lung cancer cells.^44^ Additionally, Ezzatifar et al. investigated recombinant peptides derived from the RBD of NCL to analyse serum samples from cancer patients. Their study demonstrated that anti‐RBD‐NCL autoantibodies in serum are indicative of cancer, particularly advanced lung cancer. Notably, these autoantibodies were also elevated in early‐stage lung cancer, highlighting their potential for early detection.^45^ These findings collectively underscore the pivotal role of surface NCL targeting and the detection of autoantibodies against its central RBD domain in distinguishing lung cancer patients, especially in the early stages, from healthy individuals (Figure 2).

## 4 NCL AND LUNG CANCER PROGNOSIS

Several studies have scrutinized lung cancer specimens to assess the predictive value of NCL expression alongside other cellular markers and crucial enzymes. Investigations into NSCLC tissues have shown that NCL is more abundantly expressed in cancerous tissues than in healthy counterparts, particularly from early stages, with the proportion of NCL‐positive cells increasing significantly as the cancer progresses. The extent of NCL expression has been identified as an independent variable, correlating with longer median overall survival (OS) and disease‐free survival (DFS) when lower. Conversely, higher cytoplasmic NCL levels have been associated with shorter patient survival and a poorer prognosis, whereas increased nuclear NCL aligns with better outcomes. Further studies confirmed a direct relationship between elevated cytoplasmic NCL and DNA‐PKcs, correlating with adverse patient survival in lung cancer.^13^ Additionally, the simultaneous detection of CD31, an endothelial marker, and increased NCL^+^ cells in NSCLC tissues is linked with lower DFS and higher recurrence risk, particularly in stage I patients with smaller tumours and those undergoing surgery.^12^ Contrarily, lower NCL expression in mesothelioma and LAUD is associated with distant metastasis and rapid disease progression.^46^ Moreover, a correlation was found between the accumulation of NCL and human telomerase reverse transcriptase (TERT) in the nucleoli and nuclei of cells in SCLC and basaloid carcinomas, indicative of poor differentiation, accelerated proliferation and evasion of cellular aging and death.^47^ Additionally, Figueiredo et al. proposed the potential of non‐invasive liquid biopsies to detect altered NCL expression patterns in lung cancer patients, aiding in prognosis and treatment adherence through ELISA studies.^44^ Furthermore, the presence of anti‐RBDs NCL autoantibodies correlates significantly with key clinical indicators like tumour size, stage and growth rate, highlighting their promise as robust biomarkers for tracking prognosis and disease progression in affected individuals^45^ (Figure 2).

## 5 NCL AND LUNG CANCER THERAPY

In contemporary lung cancer research, laboratory and cell‐based studies that focus on NCL and other molecular targets as potential treatments are primarily divided into two main strategic approaches: ‘*direct targeting*’ and ‘*transmission intermediary*’. These strategies involve compounds with an affinity for surface NCL, including RNAs, aptamers and peptides. *Direct targeting* approaches observe anti‐tumour effects by engaging NCL at the plasma membrane, thereby disrupting subsequent signalling cascades essential for cancer cell survival and proliferation. A prime example is the synthetic DNA aptamer AS1411, which binds to NCL with high affinity and specificity, leading to a potent antiproliferative effect on several cancer cell types. This direct interaction accentuates the importance of NCL as a functional receptor influencing key signalling pathways and cellular mechanisms pivotal in tumorigenesis. In contrast, *transmission intermediary* strategies capitalize on the presence of NCL on the cancer cell surface to facilitate the delivery of cytotoxic drugs and tumour growth inhibitors directly into cancer cells, thus offering a targeted approach to therapy. Although these new methods highlight the critical function of NCL in the development and treatment of lung cancer, it is important to mention that these studies have not progressed to human clinical trials on a global scale (Table S1).

In ‘*direct targeting*,’ strategies, natural and synthetic ligands exhibit anticancer effects by disrupting critical signalling pathways downstream of NCL (Figure 2).^48^

### 5.1 AS1411

Although lung cancer studies primarily highlight the role of AS1411 as a carrier of anti‐tumour compounds, its direct anti‐proliferative and methuosis‐inducing effects have also been reported.^41^, ^49^

### 5.2 Endostatin

Endostatin derived from the C‐terminus of collagen XVIII,^50^ is recognized for its anti‐angiogenic and anti‐tumour properties, especially in China where it is approved for treating NSCLC patients.^51^ Further investigations demonstrated that endostatin resulted in a reduction in tumour size and growth, which was significantly associated with a decrease in the number of CD31‐positive blood vessels in the orthotopic Lewis lung carcinoma (LLC) model. This was corroborated by human microvascular endothelial (HMEC) cell line studies, where endostatin inhibited endothelial cell proliferation and migration. The anti‐angiogenic effects were reversed by using an anti‐NCL antibody or shRNA knockdown of NCL, indicating that endostatin's action is mediated through NCL on blood vessels.^52^ Also, this inhibition of endothelial and osteoclast activity is facilitated through a complex with integrin α5β1, NCL and urokinase plasminogen activator receptor (uPAR), reducing tumour metastasis.^53^, ^54^ Additionally, it impedes cancer cell metastasis, invasion and angiogenesis, and modulates tumour‐infiltrating macrophages (TIMs) functions, enhancing cytotoxic effector cells' infiltration like natural killer (NK) and CD8^+^ T cells, thus boosting anti‐tumour responses.^55^, ^56^ This emphasizes that endostatin primarily acts by disrupting tumour vascularization. Building on this, E‐M, an enhanced form of endostatin, demonstrates significant anti‐angiogenic and anti‐tumour properties attributed to its increased ATPase activity. E‐M disrupts the p38/MAPK/Erk1/2 signalling pathway, thereby impairing the mobility and activation of TIMs. Importantly, E‐M utilizes the same NCL‐containing complex for cellular entry via caveolae/lipid raft‐ and clathrin‐mediated pathways, a critical mechanism for inhibiting TIM‐associated proliferation and angiogenesis in lung carcinoma cells in vitro. In NSCLC models, E‐M has shown efficacy in reducing macrophage presence and vascularization within tumours, leading to significant tumour reduction.^57^ These findings underscore E‐M's potential as a therapeutic agent in combating lung carcinoma by targeting specific cellular pathways and components.

### 5.3 Indomethacin

Indomethacin, a non‐selective non‐steroidal anti‐inflammatory drugs (NSAIDs) renowned for its pain relief and anti‐inflammatory effects, has garnered attention for its anticancer properties beyond cyclooxygenase (COX) inhibition.^58^ Recent insights by Buelvas et al. (2023) revealed that indomethacin increased levels of spermidine/spermine‐N1‐acetyltransferase‐1 (SSAT‐1), a critical enzyme in polyamine metabolism and cell cycle arrest in lung cancer cells. This elevation in SSAT‐1 occurs independently of peroxisome proliferator‐activated receptor‐γ (PPAR‐γ). Rather, it involves a decrease in NCL levels, that regulates SSAT‐1 mRNA before translation, establishing a clear link between indomethacin's effects and NCL modulation. Additionally, indomethacin inhibits cyclin‐dependent kinase 1 (CDK1), which phosphorylates NCL during mitosis, thus interfering with a vital cell proliferation pathway. The synergy of indomethacin with methoctramine, a specific inhibitor of polyamine oxidase (PAOX), significantly curtailed the proliferation of NSCLC cells.^59^ This finding offers a nuanced perspective on indomethacin's role in lung cancer therapy.

### 5.4 CAMSAP3

Calmodulin‐regulated spectrin‐associated protein 3 (CAMSAP3), plays a crucial role in maintaining cell function and architecture, particularly in stabilizing microtubules.^60^ Recent research by Seephan et al. (2023) has shown that reduced CAMSAP3 expression correlated with poor outcomes in LAUD patients, and its decrease enhanced invasive and angiogenic capabilities in NSCLC cells. Restoration of CAMSAP3 levels has been observed to counter these effects. At the molecular level, the absence of CAMSAP3 increases the activity of hypoxia‐inducible factor 1‐alpha (HIF‐1α) and its downstream targets, including VEGFA and MMPs 2 and 9, vital in promoting metastasis and angiogenesis. This process is partly due to the interaction between NCL and CAMSAP3, which governs the stability and expression of HIF‐1α mRNA. This interaction highlights the importance of the NCL/CAMSAP3 relationship in regulating aggressive metastatic and angiogenic tendencies in lung cancer cells, establishing CAMSAP3 as a key suppressor in lung cancer progression by influencing the NCL/HIF‐1α mRNA complex.^61^

### 5.5 MMP7

MMP7 plays a nuanced yet pivotal role in lung cancer progression by targeting NCL at Asp255. This interaction amplifies lung cancer growth through the upregulation of oncogenic factors like MMP9 and anaplastic lymphoma kinase (ALK), and the downregulation of tumour suppressor genes such as kruppel‐like factor 6 (KLF6). Moreover, MMP‐7 interacts with cell surface NCL to facilitate the nucleolar translocation and cleavage of p53, playing a crucial role in the survival and proliferation of lung cancer stem cells.^62^ So, strategies aimed at inhibiting MMP7 activity, thereby preventing NCL cleavage, could significantly impede the molecular pathways that facilitate lung malignancies.^63^ This approach is emerging as a promising targeted intervention to obstruct lung cancer advancement by modulating the regulatory effects of MMP7 on essential oncogenes and tumour suppressor genes.

### 5.6 P‐NCL

Phosphorylated NCL (P‐NCL), plays a crucial role in lung cancer progression, with higher levels correlating with worse patient outcomes. Studies, particularly on A549 NSCLC cells, have demonstrated that a specific missense mutation in P‐NCL (Thr76Ala) significantly reduces cell proliferation and migration. These findings suggest that targeting P‐NCL could be an effective therapeutic strategy for NSCLC by diminishing tumour growth and metastasis.^64^ By focusing on P‐NCL, we can inhibit key processes in cancer progression, underlining its importance as both a biomarker and a potential innovative treatment target.

### 5.7 LIX1L

Limb expression 1‐like protein (LIX1L), as an RNA‐binding protein, is essential for post‐transcriptional regulation and has been identified to significantly influence NSCLC progression, particularly by facilitating epithelial–mesenchymal transition (EMT), a key step in cancer metastasis. Li et al. research team underscored a strong correlation between LIX1L expression and EMT markers in NSCLC. LIX1L not only marks but also exacerbates malignant behaviours including cell migration, invasion, resistance to apoptosis (anoikis) and proliferation. Moreover, it contributes to resistance against epidermal growth factor receptor‐tyrosine kinase inhibitors (EGFR‐TKIs). The interaction of LIX1L with NCL in the nucleoli, enhancing rRNA synthesis, is vital for promoting EMT's aggressive traits in NSCLC cells. Disrupting the LIX1L‐NCL interaction, via NCL knockdown or inhibiting rRNA synthesis, has shown promise in mitigating LIX1L's malignancy‐enhancing effects. These insights suggest that targeting the LIX1L‐NCL‐rRNA pathway might offer a novel and efficacious therapeutic avenue to counteract EMT and associated drug resistance in NSCLC.^65^

### 5.8 N6L

N6L, known as a NCL antagonist, is a pseudopeptide targeting NCL, pivotal in restraining tumour growth and angiogenesis.^66^, ^67^, ^68^, ^69^, ^70^ It has been shown to reduce tumour cell invasiveness by blocking essential cell survival pathways, decelerating the cell cycle, inducing autophagy and limiting cell proliferation.^71^, ^72^, ^73^ Notably, N6L has reduced expression of L1‐ORF1p, a protein associated with cancer progression, in various NSCLC cell lines including A549, NCI‐H520, NCI‐H1299 and NCI‐H460. Its antiproliferative effects are particularly evident in cell lines with high L1‐ORFP protein levels. Moreover, N6L treatment in NSCLC tumour xenografts in nude mice has led to significant tumour reduction by downregulating L1‐ORF1p expression.^74^ These results underscore N6L's potential as a significant agent in NSCLC therapy, highlighting its effectiveness in diminishing tumour viability and disrupting vital cancer proliferation.

### 5.9 DENTAC

Dendronized DNA Chimeras (DENTAC) utilize dendritic DNA for membrane protein degradation by lysosomal transport. Zhu et al. (2023) demonstrated the efficacy of DENTAC in degrading oncogenic membrane proteins such as NCL and EGFR. In vitro experiments showed that DENTAC successfully degraded NCL in A549 cells. Additionally, in a mouse model, NCL‐targeting DENTAC significantly inhibited tumour growth, reducing tumour volume by up to 76%. These results underscore DENTAC's potential as a powerful anti‐cancer therapeutic approach.^75^

### 5.10 AS

Acharan sulfate (AS), derived from the giant African snail *Achatina fulica*, exhibits unique anti‐tumour properties without direct cytotoxicity to cancer cells. Joo et al. (2010) found that AS binds to NCL on A549 cell surfaces, causing NCL to move from the nucleus to the cytoplasm. This translocation altered growth factors like basic fibroblast growth factor (bFGF) and signalling proteins such as p38, p53 and pERK, highlighting AS‐NCL interaction's role in tumour inhibition. In vivo studies confirmed that AS inhibited tumour growth primarily through anti‐angiogenic mechanisms with minimal cytotoxicity. These findings suggest AS disrupts tumour progression by modulating cellular signalling and inducing NCL translocation.^76^

The ‘*Transmission intermediary*’, approach in lung cancer therapy uses NCL ligands to deliver cytotoxic drugs directly to tumour cells, preserving healthy lung tissue. This method enhances drug effectiveness and minimizes harm to healthy cells by ensuring precise delivery to tumours. (Figure 2).

### 5.11 Aptamers

Aptamers, particularly AS1411 has been used in various innovative ways to target lung cancer cells. For instance, it is incorporated into polyamidoamine (PAMAM) polymer derivatives to deliver Bcl‐xl shRNA plasmids to A549 lung cancer cell line, resulting in a significant decrease in target gene expression and inducing apoptosis.^77^ Additionally, AS1411‐coated polyethylene glycol‐poly(lactic‐co‐glycolic acid) nanoparticles, combined with gentamicin, have improved the delivery and bioavailability of gemcitabine, enhancing its anti‐proliferative effects.^78^ Further applications include AS1411 in pH‐sensitive chitosan nanoparticles (CSNPs) for delivering tyrosine kinase inhibitor erlotinib (AS1411‐En‐CSNPs), leading to increased apoptosis,^79^ and in theranostic probes for both detecting and targeting lung cancer cells. The aptamer's role extends to the targeted delivery of radioactive agents and chemotherapeutic drugs like methotrexate using gold nanoclusters (MTX@AuNCs‐CS‐AS1411), focusing drug action and reducing non‐specific effects.^15^, ^80^ It is also part of an advanced theranostic probe containing a miRNA‐221 molecular beacon copolymerized with magnetic fluorescence nanoparticles (MFAS miR‐221), proving effective in both detecting and targeting lung cancer cells.^81^ Additionally, AS1411‐mediated delivery of anti‐osteopontin (OPN) siRNA has shown to inhibit tumour growth in the 344SQ lung carcinoma mouse model by reducing OPN production.^82^ Similarly, another aptamer, aptNCL, conjugated with siRNA targeting SLUG (aptNCL‐SLUG) and neuropilin 1 (aptNCL‐NRP1), has been effective in suppressing transcription and downstream signalling pathways, thereby restraining tumour growth, metastasis and angiogenesis in a lung cancer mouse model while preserving healthy cells.^83^ NRP1, involved in the VEGF/PI3K/Akt signalling pathway, is known for promoting cell migration, invasion and angiogenesis in lung cancer, while SLUG is implicated in facilitating invasion, migration and epithelial–mesenchymal transition of cancer cells.^84^, ^85^, ^86^ The strategic use of aptNCL‐siRNA constructs to inhibit these pathways offers a promising approach for addressing lung cancer, ensuring a targeted therapeutic impact with minimal effects on healthy tissue.^83^ Additionally, AS1411‐tagged PEGylated liposomes containing Withaferin A (ALW) notably reduced cell viability and increased apoptosis in A549 and NCL‐H23 cells, thereby exhibiting strong anti‐tumour activity in vitro.^87^ Withaferin A is a bioactive compound derived from the plant *Withania somnifera*, known for its anti‐cancer properties. Similarly, the targeted delivery of doxorubicin (DOX) to A549 cells was achieved using pH‐responsive, AS1411‐functionalized DOX‐loaded polymeric nanoparticles, resulting in substantial tumour reduction and enhanced apoptosis in both in vitro experiments and in tumour‐bearing mice.^88^ Another study employing the same technology facilitated the co‐delivery of DOX and a FOXM1 aptamer to lung tumour cells, leading to a pronounced increase in anti‐tumour effects, reduced metastasis and decreased cell proliferation, likely due to the role of FOXM1 in mitigating chemotherapy resistance.^89^ Furthermore, Endo‐rDFN, which incorporates Endostar, an anti‐angiogenic drug, encapsulated within a reconfigurable DNA framework nanotube with AS1411 aptamers, significantly inhibited tumour growth and improved survival rates in A549 lung cancer models while minimizing systemic toxicity.^90^ Additionally, AS1411‐modified chitosan‐ss‐polyethylenimine‐urocanic acid micelles co‐delivering DOX and toll‐like receptor 4 (TLR4) siRNA exhibited excellent tumour targeting, significant anti‐tumour efficacy and enhanced survival rates in tumour‐bearing mice, with low systemic toxicity and improved tumour penetration.^91^

### 5.12 Peptides

They, including the 31‐amino acid fragment of HMGN2 protein known as F3 (KDEPQRRSARLSAKPAPPKPEPKPKAPAKK), are recognized for their potential in lung cancer therapy. Initially identified by Ruoslahti and colleagues using in vivo phage display techniques,^92^ F3 peptide is known to bind with NCL on the cell surface and then translocate to the nucleus.^93^ An innovative application involves labeling F3 peptide with technetium‐99 m to coat DOX‐loaded liposomes ((99mTc)‐F3 peptide‐DOX) for targeted delivery to bevacizumab‐resistant lung carcinoma cells, including H441, A549 and H1975. This conjugation notably enhances cellular uptake, increases cytotoxicity and potentially reduces metastatic ability of the tumour cells.^94^ Further research indicates that the F3 peptide, when combined with DOX, significantly amplifies the drug's cytotoxicity in lung carcinoma cells with varying NCL expression levels.^95^ Additionally, AGM‐330, another novel peptide binding to NCL, is being explored along with Paclitaxel (AGM‐330‐PTXL) for its antagonistic properties in lung cancer, though research into its efficacy and application continues.^96^ These insights highlight the promising role of peptides in targeting NCL to improve the delivery and effectiveness of cancer treatments, paving the way for more effective lung cancer therapies.

### 5.13 Nucleic acid‐based adjuvants: hopeful yet hypothetical

Nucleic acid‐based adjuvants, such as CpG ODNs and poly(I:C), hold promise as vaccine adjuvants for infectious diseases and cancers. These adjuvants induce Th1‐type and CD8^+^ T cell responses by activating TLRs in antigen‐presenting cells (APCs), leading to the production of pro‐inflammatory cytokines and type I interferons.^97^ In a study by Kitagawa et al. (2022), it was shown that NCL on dendritic cells (DCs) binds to CpG ODNs and poly(I:C), promoting their internalization and cytokine production, which activates antigen‐specific antibodies and T cell responses. Targeting NCL on DCs enhanced IL‐6 and IL‐12 production, crucial for activating NK cells, macrophages and other DCs.^98^ Using these adjuvants in lung cancer could enhance anti‐tumour immune responses by inducing anti‐tumour cytokines and activating effector immune cells. This process can increase the immunogenicity of tumour cells, facilitating their recognition and destruction by the immune system. Additionally, the internalization of these adjuvants into lung cancer cells via NCL may induce immunogenic cell death,^99^ leading to the release of tumour antigens. This release can further stimulate tumour‐specific immune responses, creating a dual mechanism of action: enhancing immune responses and directly targeting tumour cells. Although these mechanisms are promising, they require extensive experimental validation in lung cancer. Continued research is anticipated to provide significant insights and potentially develop effective therapeutic strategies for lung tumours.

## 6 CONCLUDING REMARKS

Despite the somewhat limited data, evidence suggests that extranuclear NCL, particularly its surface expressions, and anti‐RBD autoantibody levels could have significant prognostic value in predicting DFS, tumour growth, proliferation and metastasis in certain lung tumour types, especially in early stages. However, the prognostic value of NCL varies and must be carefully considered in a disease‐specific context to accurately predict lung cancer progression. The use of radioactive and fluorescent labels, along with nano‐sized ligands such as aptamers and pseudopeptides targeting NCL, significantly enhances the precision and likelihood of detecting lung cancer cells. The therapeutic potential of natural NCL ligands like endostatin is particularly compelling and may be integrated into future lung cancer treatments. Additionally, some ligands, including aptamers, not only exhibit direct antitumor properties but also serve as efficient vectors for delivering cytotoxic agents to cancer cells. The exploration of other potential anticancer ligands, such as kallistatin, DENTAC and AS, targeting surface NCL in various cancers, presents a clear opportunity to evaluate their effectiveness in lung cancer specifically. Moving forward, transitioning this laboratory and experimental evidence into human studies and clinical trials will be crucial to address the safety and efficacy concerns associated with these potential treatments.

## AUTHOR CONTRIBUTIONS

**Amirabbas Rostami:** Writing – original draft (equal). **Ehsan Ranjbar:** Writing – original draft (equal). **Somayeh Amiri:** Conceptualization (equal); visualization (equal). **Fatemeh Ezzatifar:** Conceptualization (equal); writing – review and editing (equal).

## FUNDING INFORMATION

The authors declare that no funds, grants or other support were received during the preparation of this manuscript.

## CONFLICT OF INTEREST STATEMENT

The authors declare no conflict of interest.

## CONSENT FOR PUBLICATION

All authors are aware of the content of this paper and agree to publish.

## Supporting information


Table S1.

## Data Availability

Data sharing not applicable to this article as no datasets were generated or analysed during the current study.
