# Supplementary material for: Beyond biomarkers: Exploring the diverse potential of a novel phosphoprotein in lung cancer management
Source: J Cell Mol Med. 2024 Sep 20;28(18):e70077. doi: 10.1111/jcmm.70077 (PMC11415302; doi:10.1111/jcmm.70077)
Supplement: Supplementary file 1 — Table S1. [file JCMM-28-e70077-s001.docx]

Supplementary information

**Table S1:** Nucleolin (NCL) targeting agents and their mechanisms in lung cancer therapy.

| Therapeutic strategy | Mechanism of action | Pre-clinical stage | Reference |
| --- | --- | --- | --- |
| *Direct targeting* | | | |
| AS1411 | Reduced tumor growth; increased macropinocytosis and methuosis; elevated Rac1 and EGFR^ǂ^ activation | *In vitro* | ^41^ |
| Endostatin | Reduced angiogenesis and tumor growth; inhibited critical phosphorylation  Reduced tumor size and blood vessel density in xenograft mice model | *In vitro*  *In vivo* | ^52^ |
| Indomethacin | Increased the SSAT-1^ǂ^ level; cell cycle arrest, inhibited the CDK1^ǂ^ activity and cell proliferation | *In vitro* | ^59^ |
| CAMSAP3 inhibition^ǂ, a^ | Reduced cell invasion; downregulation of MMP2^ǂ^ and MMP9 expression; reduced angiogenesis and VEGFA^ǂ^  Reduced metastasis and angiogenesis in mice model | *In vitro*  *In vivo* | ^61^ |
| E-M^ǂ^ | Reduced TAM^ǂ^ motility; blocked macrophage recruitment and polarization; suppressed proangiogenic effects  Inhibited macrophage recruitment and tumor angiogenesis | *In vitro*  *In vivo* | ^57^ |
| MMP7 deactivation^b^ | Reduced cell proliferation and MMP9 levels; shorten MMP9 mRNA stability  Blocked tumor growth and metastasis; reduced MMP9 level in mice model | *In vitro*  *In vivo* | ^63^ |
| Mutant P-NCL^ǂ^ | Decreased P-NCL, cell proliferation and migration | *In vitro* | ^64^ |
| LIX1L inhibition^ǂ, a^ | Reduced ribosomal RNA synthesis and nucleolar size; blocked EMT^ǂ^; disturbed ribosome biogenesis | *In vitro* | ^65^ |
| N6L | Reduced cell viability; increased apoptosis; activated p53 pathway; decreased NPM1^ǂ^, nucleolin and fibrillarin | *In vitro* | ^74^ |
| DENTAC^ǂ^   \|  \| \| --- \| | Reduced tumor growth; enhanced tumor apoptosis  Reduced tumor growth; increased survival rate; no significant toxicity | *In vitro*  *In vivo* | ^75^ |
| AS^ǂ^ | Indirect cytotoxicity: limited cell growth inhibition, changes in growth factors and signaling proteins  Reduced tumor growth and angiogenesis; no significant toxicity | *In vitro*  *In vivo* | ^76^ |
| *Transmission intermediary* | | | |
| Aptamers | | | |
| Modified PAMAM^ǂ^/ Bcl-xL shRNA-plasmid/AS1411 | Increased transfection efficiency; efficient delivery; induced apoptosis | *In vitro* | ^77^ |
| AS1411-GEM-NPs^ǂ^ | Enhanced cellular uptake and cytotoxicity; controlled release | *In vitro* | ^78^ |
| AS1411-En-  CSNPs^ǂ^ | Enhanced cellular uptake; pH-dependent drug release; cytotoxicity; apoptosis induction | *In vitro* | ^79^ |
| ^57^Co-DOTA-AS1411^ǂ^ | Efficient uptake by cancer cells; increase in drug specific uptake  Reduced tumor growth and metastasis | *In vitro*  *In vivo* | ^15^ |
| MTX@AuNCs-CS-AS1411^ǂ^ | Enhanced anticancer activity; selective uptake  Tumor accumulation and growth inhibition; no overt toxicity in mice model | *In vitro*  *In vivo* | ^80^ |
| MFAS miR-221 MB^ǂ^ | Reduced cell viability  Reduced tumor size | *In vitro*  *In vivo* | ^81^ |
| aptNCL-OPN^ǂ^ siRNA | Reduced OPN mRNA and protein levels  Inhibited tumor growth | *In vitro*  *In vivo* | ^82^ |
| aptNCL-SLUG siR^ǂ^ and aptNCL-NRP1 siR^ǂ^ | Synergistic inhibition of cell migration and invasion; decreased filopodia formation; dose-dependent tumor suppression  Suppressed tumor growth; reduced CTCs^ǂ^; decreased micro vessel formation; no significant toxicity | *In vitro*  *In vivo* | ^83^ |
| ALW^ǂ^ | Reduced cell viability and anti-apoptotic gene expression; increased apoptosis, pro-apoptotic gene expression and cellular uptake | *In vitro* | ^87^ |
| APT-DOX-PLGA-PVP NPs^ǂ^ | Reduced cell viability; enhanced DOX cytotoxicity, cellular uptake, oxidative stress and apoptosis  Reduced tumor size and weight loss; no significant toxicity; improved overall health and survival | *In vitro*  *In vivo* | ^88^ |
| Dox-DNA Nanostructure Complex | Reduced cell viability; no significant toxicity | *In vitro* | ^89^ |
| Endo-rDFN^ǂ^ | Reduced cell proliferation and migration  Reduced tumor growth; no weight loss; reduced proliferation and angiogenesis markers; increased survival rate; enhanced anti-tumor activity | *In vitro*  *In vivo* | ^90^ |
| ACPU^ǂ^ | Enhanced cytotoxicity and tumor penetration; reduced migration and invasion  Reduced tumor growth and systemic toxicity; enhanced antitumor efficacy, survival rates and drug delivery | *In vitro*  *In vivo* | ^91^ |
| Peptides | | | |
| (99mTc)-F3 peptide-DOX^ǂ^ | Increased cytotoxic activity of DOX | *In vitro* | ^94^ |
| AGM-330-PTX^ǂ^ | Binding specificity; cell growth inhibition; target protein identification; NCL neutralization  Enhanced specificity of targeting and therapeutic efficacy of PTX; good stability | *In vitro*  *In vivo* | ^96^ |
| Nucleic acid-based adjuvants | | | |
| B-type CpG ODNs^ǂ^, A-type CpG ODNs and Poly(I:C) | Enhanced adjuvant uptake and human/mouse DC^ǂ^ activation via NCL; Induced cytokine production and expression of co-stimulatory molecules  Enhanced IL-12 p40 production, adjuvant distribution and immune response in DCs and macrophages | *In vitro*  *In vivo* | ^100^ |

^ǂ^EGFR: Epidermal Growth Factor Receptor, SSAT-1: Spermidine/Spermine N1-Acetyltransferase-1, CDK1: Cyclin-Dependent Kinase 1, CAMSAP3: Calmodulin Regulated Spectrin-Associated Protein Family Member 3, MMP: Matrix Metalloproteinase, VEGFA: Vascular Endothelial Growth Factor A, E-M: Synthetic Endostatin Enhanced in ATPase Activity, TAM: Tumor Associated Macrophage, P-NCL: Phosphorylated NCL, LIX1L: Limb Expression 1-Like, EMT: Epithelial-Mesenchymal Transition, NPM1: Nucleophosmin1, DENTAC**:** DNA-Encoded Nucleolin-Targeting Anticancer Compound, AS: Acharan Sulfate, PAMAM: Polyamidoamine, GEM-NPs: Gemcitabine Nanoparticles, AS1411-En-CSNPs: AS141-Erlotinib-Chitosan Nanoparticles, ^57^Co-DOTA-AS1411: Cobalt-57-1,4,7,10-tetraazacyclododecane-1,4,7,10-tetraacetic acid- AS1411, MTX@AuNCs-CS-AS1411: Methotrexate-Gold Nanoclusters- Chitosan- AS1411, MFAS miR-221 MB: Magnetic-Fluorescent Nanoparticles-AS1411-microRNA-221 Molecular Beacon, OPN: Osteopontin, aptNCL-SLUG/ NRP1 siRs: NCL aptamer-siRNAs targeting SLUG and NRP1 genes, CTC: Circulating Tumor Cell, ALW: AS1411 Functionalized Withaferin A Loaded PEGylated Nanoliposomes, APT-DOX-PLGA-PVP NPs: Aptamer Functionalized Doxorubicin Loaded Poly (D, L-lactic-co-glycolic acid), Poly (N-vinylpyrrolidone) Nanoparticles, Endo-rDFN: Endostar-reconfigurable DNA Framework Nanotube, ACPU: AS1411-Aptamer-Modified Chitosan-ss-Polyethylenimine-Urocanic Acid, (99mTc)-F3 peptide-DOX: Technetium-99m-F3 Peptide-Doxorubicin, AGM-330-PTX: AGM-330-Paclitaxel, ODNs: Oligodeoxynucleotides, DC: Dendritic Cell.

^a^CAMSAP3 and LIX1L inhibitors are not yet commercially available.

^b^Potential MMP-7 inhibitors, yet to be confirmed effective for lung cancer, include Marimastat, Prinomastat, Minocycline, Tetracycline derivatives, Natural Compounds, Polyphenols, and Small Molecule Inhibitors.
